# Self-reported knee function and activity level are reduced after primary or additional anterior cruciate ligament injury in female football players: a five-year follow-up study

**DOI:** 10.1016/j.bjpt.2023.100573

**Published:** 2023-11-22

**Authors:** Anne Fältström, Martin Hägglund, Henrik Hedevik, Joanna Kvist

**Affiliations:** a Rehabilitation Centre, Ryhov County Hospital, Jönköping, Region Jönköping County, Sweden; b Unit of Physical Therapy, Department of Health, Medicine and Caring Sciences, Linköping University, Linköping, Sweden; c Stockholm Sports Trauma Research Center, FIFA Medical Centre of Excellence, Karolinska Institute, Stockholm, Sweden

**Keywords:** Knee, Return to sports, Reinjuries, Satisfaction, Soccer

## Abstract

**Background:**

Playing football involves a high risk of anterior cruciate ligament (ACL) injuries and these may affect knee function and activity level.

**Objectives:**

To measure changes in self-reported knee function, activity level, and satisfaction with knee function and activity level in female football players with or without an ACL-reconstructed knee.

**Methods:**

Female football players, age 19.9 (SD 2.6) years, with either a primary ACL-reconstruction 1.6 (SD 0.7) years after ACL-reconstruction (*n* = 186) or no ACL injury (*n* = 113) were followed prospectively for five years. Self-reported data collected at baseline and follow-up included knee function (International Knee Documentation Committee Subjective Knee Form [IKDC-SKF]), activity level (Tegner Activity Scale), and satisfaction with knee function (Likert scale 1=happy; 7=unhappy) and activity level (1–10 scale). Information on any new ACL injury during the follow-up period was collected.

**Results:**

Players with ACL-reconstruction at baseline who either did (*n* = 56) or did not (*n* = 130) sustain an additional ACL injury, and players with no injury at baseline who remained injury free (*n* = 101) had a lower Tegner score at follow-up. Players with additional ACL injury had lower IKDC-SKF score (mean difference: −11.4, 95% CI: −16.0, −6.7), and satisfaction with activity level (mean difference: −1.5, 95% CI: −2.3, −0.7) at follow-up. Players with no additional ACL injury had higher satisfaction with knee function (mean difference: 0.6, 95% CI: 0.3, 0.9) at follow-up. Players with no ACL injury had lower satisfaction with activity level (mean difference: −0.7, 95% CI: −1.1, −0.3) at follow-up. Players with additional ACL injury had larger decreases in all variables measured compared to the two other groups.

**Conclusion:**

Primary, and even more so additional, ACL injuries decreased self-reported knee function, activity level, and satisfaction with knee function and activity level in female football players.

## Highlights

• ACL injuries decrease satisfaction, knee function, and activity level.
• Activity decreases with time, and more so in players who sustain an ACL injury.
• Players with additional ACL injuries had the largest decrease in all variables.

## Introduction

Anterior cruciate ligament (ACL) injury is a severe knee injury, where female athletes have a double incidence rate compared to male athletes.^1^ ACL injuries often lead to surgical treatment, especially for young athletes with the goal to return to sport.^2^ However, returning to pivoting sports after ACL reconstruction (ACLR) involves a high risk of new knee injuries; 42% of female football players who returned to football after ACLR sustained a second ACL injury.^3^ Still, females who returned to football have higher ratings for self-reported knee function and knee-related quality of life compared to those who did not return to football.^4^

Factors associated with self-reported knee function and activity level have been evaluated at various time points after ACLR.^5^^,^^6^ Predictors of lower self-reported knee outcome scores include quadriceps weakness,^7^ concomitant injury at the time of ACLR, revision surgery, lower baseline scores in the International Knee Documentation Committee Subjective Knee Form (IKDC-SKF), the Knee injury and Osteoarthritis Outcome Score, and Marx activity rating scale, higher body mass index, lower level of education, smoking, and use of allografts.^5^^,^^6^ Predictors for lower activity level after ACLR include severe medial cartilage injury,^5^ female sex, and revision surgery.^6^ There are few studies comparing self-reported outcomes and activity level between patients with primary ACLR and patients with additional ACLR (revision or ACLR in the contralateral knee),^8^, ^9^, ^10^ and there is a lack of studies on patients who sustain an additional ACL injury treated without ACLR. The design in previous studies is most often cross-sectional comparing different self-reported outcomes, with no comparisons to knee-healthy controls, and with limited information about patients’ satisfaction with knee function and activity level.^8^, ^9^, ^10^ By using a prospective design, it is possible to measure changes in self-reported knee function, activity level, and satisfaction with knee function and activity level before and after the injury. Thus, long-term prospective studies reporting self-reported outcomes after primary and additional ACL injury compared with knee-healthy controls in a high-risk group of female football players are lacking.

The aims of this study were to: (1) measure changes in self-reported knee function, activity level, and satisfaction with knee function and activity level from baseline to five years post baseline assessment; (2) compare the changes between three different groups of female football players: players with a primary ACLR at baseline (6–36 months after reconstruction) who sustained a new ACL injury during follow-up, players with a primary ACLR at baseline who did not sustain an ACL injury during follow-up, and players who had no ACL injury at baseline or during the follow-up. Our hypothesis was that players with a primary ACLR who sustained a new ACL injury would report a greater decrease in knee function, activity level, and satisfaction with knee function and activity level compared with players with a primary ACLR who did not sustain a new ACL injury and players with no ACL injury. A second hypothesis was that players with no ACL injury would have the smallest change in the studied variables.

## Methods

### Study design

This was an exploratory analysis of a prospective cohort study. Short-term (two-year follow-up) descriptive data of new knee injuries, knee function, and activity level outcomes have been published previously for 117 players with ACLR (111 included in the current study) and for 119 knee-healthy controls (113 included in the current study).^11^ Outcomes from 5 to 10 years after ACLR regarding new knee injuries have also been published for this cohort.^3^

### Participants

**Fig. 1:**
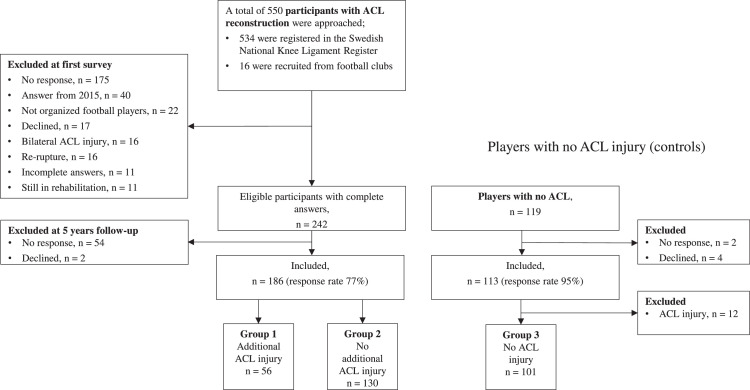
Flowchart for the selection of participants. Players with complete answers at the first survey (study baseline) and at follow-up were included. Twelve players in the control group sustained an anterior cruciate ligament (ACL) injury between follow-up time points and were therefore not included in the final analysis. In the main study (prospective cohort study), the purpose was to find active football players for the ongoing prospective study about risk factors and therefore 40 players (in 2015) who had quit football did not answer the total survey.

Females with primary unilateral ACLR (6–36 months prior to study inclusion) were identified via the Swedish National Knee Ligament Register (SNKLR)^12^ and via advertising on three regional football district websites. Exclusion criteria were having a fracture, an associated posterior cruciate ligament injury, and/or surgically treated injuries to either the medial or lateral collateral ligament. Females aged 16–25 years who had injured their knee when playing football were invited to participate in the study. A survey was sent at the football pre-season (January–April) in 2013–2015 to 534 potentially identified eligible participants in the SNKLR, of which 226 were eligible participants. An additional 16 active players were recruited via advertisements. 186 of the total 242 eligible participants answered the survey both at study baseline and at follow-up and were included in the current study (Fig. 1). A control group of 119 female players with no ACL injury, recruited from the same teams as players with ACLR and matched regarding age and playing position was included to establish the normal course of a female football player's activity level^13^ and reported satisfaction of knee function and activity level.^11^

The study was approved by the Swedish Ethical Review Authority (Dnr 2012/24–31, 2013/75–32 and 2020–01093) and the SNKLR board. The study was conducted in accordance with the code of ethics of the World Medical Association. All players received written information about the study and gave written consent.

### Procedures

Players received a questionnaire about background data including demographics, football participation, and football-related factors if applicable (preferred kicking leg, level of play, most important reason for playing football if applicable, use of orthosis, and risk behavior with four predefined choices (“avoid risks at any price”, “try to avoid risks most of the time”, “sometimes take deliberate risks”, “often take deliberate risks”). Data such as age, surgical data, and associated injuries were collected from the SNKLR.

Players with ACLR reported knee function by completing the IKDC-SKF with a score ranging from 0 (worst) to 100 (best).^14^^,^^15^ IKDC-SKF is developed for persons with knee injuries, hence players with no ACL injury did not answer the questionnaire. IKDC-SKF is valid, test-retest reliable, and responsive for change.^14^, ^15^, ^16^ The players stated their activity, level of activity (elite, competitive or recreational), and times/week the activities were performed. Then, the activity level was graded for all players by the first author according to the Tegner Activity Scale,^8^^,^^17^ which assesses activity level on a scale from 0 to 10, where 0 corresponds to sick leave due to knee problems and 10 corresponds to participation in football at national level.^17^ The Tegner activity scale has acceptable psychometric parameters (internal consistency, test-retest reliability, criterion validity, construct validity, floor and ceiling effects, and responsiveness).^18^ Satisfaction with knee function was measured with the question: “If you had to live with your current knee function for the rest of your life just the way it has been in the last week, would you feel…?”, with response options ranging from 1 to 7: happy (1), satisfied, mostly satisfied, mixed, mostly dissatisfied, dissatisfied, and unhappy (7).^8^^,^^19^^,^^20^ The question is reliable and valid in patients with low back pain^19^ and valid for patients with ACLR.^20^ Players also rated their satisfaction with their current activity level on a scale ranging from 1 (not satisfied at all) to 10 (very satisfied).^8^^,^^21^

The follow-up was 5 years after baseline. The players answered the same baseline questions using a web-based survey. Nonresponders were sent up to four reminders. They also answered the question “Have you sustained any (new) ACL injury?” All new ACL injuries reported by the players, whether the injury was reconstructed or not, were confirmed via the SNKLR or medical records. All players who still played football also answered a question on whether they performed any knee injury prevention program.

### Data analysis

All statistical analyses were performed in SPSS Statistics for Windows (v 27.0; IBM). Mean and standard deviation (SD) or median and interquartile range (IQR) were calculated for descriptive data. Paired samples *t*-test was used to compare within-group change from baseline to follow-up in IKDC-SKF, activity level according to the Tegner Activity Scale, and satisfaction with knee function and activity level. One-way ANCOVA (adjusted for mean baseline values of each outcome in the total cohort because of differences in scores between the three groups at baseline) was used for between-group comparisons of within-group change from baseline to the follow-up. As a sensitivity analysis we conducted the same analysis on each outcome measure, with eight players removed due to them sustaining additional ACL injury or ACLR less than one year prior to follow-up. Bonferroni correction was used for all pairwise comparisons to consider the familywise error rate. Chi-squared test was used to compare the number of players in each group still playing football at baseline and follow-up (players with a primary ACLR who sustained a new ACL injury vs players with a primary ACLR who did not sustain a new ACL injury). A nonresponse analysis was performed to evaluate potential attrition bias with Student's *t-*test (age and body mass index [BMI]) and chi-squared test (graft, presence of concomitant injuries at primary ACLR, and additional ACLR registered in SNKLR). The significance level was set at 0.05.

## Results

Of the 242 eligible players with primary ACLR at baseline, 186 responded to the follow-up survey (response rate 77%) and 56 did not respond, at a mean 6.5 (SD 1.0) years (range, 5.0–9.9 years) after their primary ACLR. At primary ACLR, hamstrings grafts (all autografts) were used in 98%, and concomitant injuries on meniscus and/or cartilage were present in 38%, of players with a primary ACLR who sustained a new ACL injury and 36% of players with a primary ACLR who did not sustain a new ACL injury. Time between study baseline and follow-up was 4.9 (SD 0.7) years.

### Group characteristics

At follow-up, 56 of the players with ACLR at baseline who sustained a new ACL injury (30%) had sustained 58 additional ACL injuries (39 graft-ruptures [66%] of which 20 [51%] were reconstructed; 19 contralateral ruptures [34%] of which 17 [89%] were reconstructed). Two of the 56 players sustained both a contralateral rupture and re-rupture, hence 58 total additional ACL injuries. Time from the additional ACL injury or ACLR to responding to the follow-up questionnaire was 38 (18) months (2–81 months). Players with a primary ACLR who did not sustain a new ACL injury included 130 players (70%).

Of the 119 players with no ACL injury at baseline, 113 responded (response rate 95%), age 24.5 (SD 2.6) years. Time between baseline and follow-up was 5.0 (SD 0.7) years. Eleven players sustained an ACL injury and one player (total 12/113; 11%) sustained two ACL injuries and were excluded in the final analysis to keep an injury-free control group (Fig. 1). Thus, players with no ACL injury included 101 players (89%) (Table 1).

**Table 1:** Demographic and football-related factors at baseline and at five-year follow-up for female football players.

Information available for all players	Players with primary ACL reconstruction at baseline (*n* = 186)					
	Group with additional ACL injury (*n* = 56)		Group with no additional ACL injury (*n* = 130)		Group with no ACL injury (*n* = 101)	
	Baseline	Follow-up	Baseline	Follow-up	Baseline	Follow-up
Age, years, mean (SD)	20.0 (2.7)	24.9 (2.7)	20.3 (2.7)	25.2 (2.8)	19.5 (2.6)	24.5 (2.7)
Body mass index, kg/m^2^, mean (SD)	22.3 (2.2)	22.9 (2.6)	22.6 (2.3)	23.3 (2.6)	22.1 (2.0)	22.7 (2.5)
Time from ACLR, months, mean (SD)	19.1 (8.3)	37.9 (18.3)^a^	19.7 (8.4)	78.4 (12.3)	NA	NA
<9 months	5 (9)	7 (12)^a^	6 (5)	0 (0)	NA	NA
9–11 months	10 (18)	1 (2)^a^	24 (18)	0 (0)	NA	NA
12–24 months	23 (41)	7 (12)^a^	53 (41)	0 (0)	NA	NA
>24 months	18 (32)	41 (73)^a^	47 (36)	130 (100)	NA	NA
Occupation						
Worker	21 (38)	32 (57)	37 (28)	94 (72)	25 (25)	70 (69)
Student	35 (63)	24 (43)	93 (72)	36 (28)	76 (75)	31 (31)
Other training (not football)	34 (61)	39 (70)	90 (69)	85 (65)	53 (52)	60 (59)

Information available only for players still playing football	*n* = 43 (77%)	*n* = 8 (14%)	*n* = 73 (56%)	*n* = 38 (29%)	*n* = 101 (100%)	*n* = 47 (47%)

**Playing position**^b^						
Goalkeeper	1 (2)	0 (0)	4 (6)	2 (5)	4 (4)	3 (6)
Defender	10 (23)	2 (25)	30 (42)	14 (37)	41 (41)	17 (36)
Midfield	25 (58)	4 (50)	25 (35)	14 (37)	40 (40)	18 (38)
Forward	7 (16)	2 (25)	13 (18)	8 (21)	16 (16)	9 (19)
**Dominant leg (preferred kicking leg)**						
Right	39 (91)	7 (88)	66 (90)	35 (92)	98 (97)	45 (96)
Left	4 (9)	1 (12)	7 (10)	3 (8)	3 (3)	2 (4)
**Level of play**						
Elite (2 top divisions)	7 (16)	4 (50)	6 (8)	5 (13)	11 (11)	8 (17)
3rd to 5th division	31 (72)	4 (50)	59 (81)	30 (79)	78 (77)	34 (72)
Lowest divisions	5 (11)	0 (0)	8 (11)	3 (8)	12 (12)	5 (11)
**Level of play compared with before the ACL injury**						
Same level	25 (60)	2 (25)	43 (60)	16 (42)	NA	NA
Higher level	9 (21)	3 (38)	12 (17)	11 (29)	NA	NA
Lower level	8 (19)	3 (38)	17 (24)	11 (29)	NA	NA
**Perceived impact of ACL injury on football playing ability**						
Same as before the injury	17 (39)	5 (63)	23 (32)	17 (45)	NA	NA
Changed playing style to control the knee	9 (21)	1 (12)	16 (22)	8 (21)	NA	NA
Use knee orthosis to be able to play	2 (5)	1 (12)	1 (1)	4 (10)	NA	NA
I continue to play despite instability or pain	1 (2)	0 (0)	3 (4)	0 (0)	NA	NA
I stop playing when I experience knee symptoms	2 (5)	0 (0)	4 (5)	1 (3)	NA	NA
I am more cautious in my playing to avoid a new injury	10 (23)	1 (12)	22 (29)	7 (18)	NA	NA
Other	2 (5)	0 (0)	4 (5)	1 (3)	NA	NA
**Knee injury prevention training with the team**						
Yes, every training	NA	3 (38)	NA	9 (24)	NA	11 (23)
Yes, 1–2 training sessions/week	NA	2 (25)	NA	7 (18)	NA	9 (19)
Yes, 1–2 training sessions/month	NA	1 (12)	NA	4 (11)	NA	9 (19)
No, but I practice by myself	NA	1 (12)	NA	9 (24)	NA	5 (11)
No, none	NA	1 (12)	NA	9 (24)	NA	13 (28)
**Most important reason for playing football**						
To win	7 (17)	2 (25)	8 (11)	2 (5)	9 (9)	5 (11)
Practice/prepare for competition	8 (19)	1 (12)	16 (22)	6 (16)	18 (18)	3 (6)
Have fun	23 (55)	5 (63)	41 (57)	26 (68)	60 (59)	31 (66)
Help the team/health reasons/other	4 (10)	0 (0)	8 (11)	4 (11)	14 (14)	8 (17)
**Risk behaviour**						
Avoided risks at any price	6 (14)	0 (0)	10 (14)	0 (0)	0 (0)	0 (0)
Tried to avoid risks most of the time	24 (57)	6 (75)	35 (50)	16 (42)	41 (41)	23 (49)
Sometimes took deliberate risks	12 (29)	1 (13)	19 (26)	17 (45)	50 (50)	16 (34)
Often took deliberate risks	0 (0)	1 (13)	8 (11)	5 (14)	10 (10)	8 (17)
Use knee brace	6 (12)	1 (13)	19 (26)	8 (21)	2 (2)	2 (4)
**Football-specific questions**						
I feel limited when playing football after the ACL injury	23 (55)	2 (25)	39 (54)	8 (21)	NA	NA
I cannot perform at the same level as before the ACL injury when playing football	19 (45)	3 (38)	47 (65)	13 (34)	NA	NA

Values are reported as n (%) if not otherwise stated. ACL, anterior cruciate ligament; ACLR, ACL reconstruction; FU, Follow-up; NA, not applicable.

a Time from additional ACL injury or ACLR to follow-up.

b One missing answer in playing position at baseline in group 2.

At baseline, 77% of the players with a primary ACLR who sustained a new ACL injury still played football compared with 56% in players with a primary ACLR who did not sustain a new ACL injury (*p* = 0.008). At follow-up, 14% in players with a primary ACLR who sustained a new ACL injury and 29% in players with a primary ACLR who did not sustain a new ACL injury still played football (*p* = 0.030) (Table 1).

### Did not reply to invitation to participate

According to the SNKLR, those who did not reply at baseline (n = 56) did not differ significantly from those who replied (n = 186) regarding age, BMI, graft, presence of concomitant injuries at primary ACLR, or additional ACLR (*p* > 0.05).

### Changes from baseline to follow-up

#### Within-group results

From baseline to follow-up, the mean IKDC-SKF score decreased among players with a primary ACLR who sustained a new ACL injury (mean difference: −11.4, 95% CI: −16.0, −6.7), while no change was seen for players with a primary ACLR who did not sustain a new ACL injury. The mean score on the Tegner Activity Scale decreased in all three groups (Table 2). The mean satisfaction with knee function increased slightly among players with a primary ACLR who did not sustain a new ACL injury (mean difference: 0.6, 95% CI: 0.3, 0.9). Satisfaction with activity level was significantly decreased among players with a primary ACLR who sustained a new ACL injury (mean difference: −1.5, 95% CI: −2.3, −0.7) and in players with no ACL injury (mean difference: −0.7, 95% CI: −1.1, −0.3) (Table 2).

**Table 2:** Changes in self-reported knee function, activity level, and satisfaction with knee function and activity level at baseline and at follow-up in female football players with ACL reconstruction at baseline who either sustained (group 1, n = 56) or did not sustain (group 2, n = 130) an additional ACL injury between follow-up time points, as well as players with no ACL injury (group 3, n = 101).

Group	Baseline, mean (95% CI)	Follow-up, mean (95% CI)	Within-group change
			Mean difference (95% CI)
**IKDC-SKF (0–100)**^a^			
1. Additional ACL injury	81.7 (78.3, 85.1)	70.4 (65.4, 75.4)	−11.4 (−16.0, −6.7)*
2. No additional ACL injury	79.2 (76.8, 81.7)	81.3 (78.7, 83.8)	2.0 (−0.2, 4.2)
Between-group difference^b^			
1–2			−12.5 (−16.7, −8.3)*
**Tegner Activity Scale (0–10)**			
1. Additional ACL injury	8.1 (7.5, 8.7)	4.0 (3.2, 4.7)	−4.1 (−5.0, −3.3)*
2. No additional ACL injury	6.4 (5.9, 7.0)	4.7 (4.2, 5.3)	−1.7 (−2.3, −1.2)*
3. No ACL injury	9.1 (9.0, 9.2)	6.0 (5.3, 6.6)	−3.1 (−3.8, −2.5)*
Between-group difference^b^ with Bonferroni correction			
1–2			−1.5 (−2.6, −0.3)*
1–3			−1.6 (−2.7, −0.4)*
2–3			−0.1 (−1.1, 0.9)
**Satisfaction with current knee function (1–7)**			
1. Additional ACL injury	3.0 (2.6, 3.4)	3.4 (2.9, 3.8)	−0.3 (−0.9, 0.2)
2. No additional ACL injury	3.0 (2.8, 3.3)	2.5 (2.2, 2.7)	0.6 (0.3, 0.9)*
3. No ACL injury	1.4 (1.2, 1.6)	1.4 (1.2, 1.6)	0.0 (−0.2, 0.3)
Between-group difference^b^ with Bonferroni correction			
1–2			−0.9 (−1.4, −0.4)*
1–3			−1.5 (−2.0, −0.9)*
2–3			−0.6 (−1.0, −0.1)*
**Satisfaction with current activity level (1–10)**			
1. Additional ACL injury	7.0 (6.3, 7.6)	5.5 (4.7, 6.3)	−1.5 (−2.3, −0.7)*
2. No additional ACL injury	6.3 (5.9, 6.7)	6.5 (6.0, 6.9)	0.2 (−0.3, 0.6)
3. No ACL injury	7.4 (7.0, 7.7)	6.7 (6.2, 7.1)	−0.7 (−1.1, −0.3)*
Between-group difference^b^ with Bonferroni correction			
1–2			−1.3 (−2.2, −0.4)*
1–3			−1 (−1.9, −0.1)*
2–3			0.3 (−0.4, 1.1)

a No baseline or follow-up values for IKDC-SKF for the players with no ACL injury are available.

b Adjusted for mean baseline values of each outcome in the total cohort.

⁎ *p* < 0.05 ACL, anterior cruciate ligament; IKDC-SKF, International Knee Documentation Committee Subjective Knee Form.

#### Between-group results

Between-group differences in within-group change from baseline to follow-up revealed that players with a primary ACLR who sustained a new ACL injury had larger decreases in knee function (IKDC-SKF) (mean difference: −12.5, 95% CI: −16.7, −8.3), activity level (mean difference: −1.5, 95% CI: −2.6, −0.3), and satisfaction with knee function (mean difference: −0.9, 95% CI: −1.4, −0.4) and activity level (mean difference: −1.3, CI: −2.2, −0.4) than players with a primary ACLR who did not sustain a new ACL injury. Compared to players with no ACL injury, players with a primary ACLR who sustained a new ACL injury had larger decreases in activity level (mean difference: −1.6, 95% CI: −2.7, −0.4) and satisfaction with knee function (mean difference: −1.5, 95% CI: −2.0, −0.9) and activity level (mean difference: −1.0, 95% CI: −1.9, −0.1)(Table 2).

### Sensitivity analysis

For the sensitivity analysis, eight players, who either had an additional ACL injury or ACLR within one year from follow-up (Table 1), were removed from the analyses. The main conclusions from the within-group and between-group comparisons did not change, but excluding these eight players generally resulted in lower change scores for the included outcomes. The main difference after excluding the eight players with a primary ACLR who sustained a new ACL injury was in IKDC-SKF (within-group change; mean difference: −7.6, 95% CI: −12.0, −3.2 compared to −11.4, 95% CI: −16.0, −6.7 and between-group change; mean difference −8.6, 95% CI: −13.1, −4.1 compared to −12.5, 95% CI: −16.7, −8.3). All other differences in change scores were small to trivial.

## Discussion

The main findings were that female football players with previous ACLR who sustained an additional ACL injury showed a large decrease in self-reported knee function, activity level, and satisfaction with knee function and activity level at five-year follow-up. All players had a notable decreased in activity level. Thus, our hypothesis was partly confirmed. Knee-healthy players also decreased their activity level and satisfaction with their activity level and these changes were consistent with players with primary ACLR at baseline who did not sustain an additional ACL injury.

Players with a primary ACLR who sustained a new ACL injury had a decrease in knee function measured with IKDC-SKF (mean difference: −11.4, 95% CI: −16.0, −6.7) compared with players with a primary ACLR who did not sustain a new ACL injury (mean difference: 2.0, 95% CI: −0.2, 4.2), who did not change. This is consistent with previous results showing a negative impact regarding self-reported knee function measured with IKDC-SKF, Lysholm Score, or the KOOS after revision surgery compared with after primary ACLR.^22^, ^23^, ^24^ Patients with additional ACL injuries probably have decreased self-reported knee function compared with patients with primary ACLR due to an additional trauma to the knee. Many of them also have additional ACLRs and other concomitant injuries to cartilage and the menisci^22^ and tibiofemoral osteoarthritis.^23^

Most of the previous studies reporting self-reported knee function includes only patients with a second ACLR and report either patients with revision or contralateral ACLR. In our cohort, we included all players with an additional ACL injury regardless of whether they had a new ACL injury in the same (graft rupture) or the contralateral knee and regardless of the treatment strategy (ACLR or not). It was more common that our female football players with an additional ACL injury underwent a second contralateral compared with ipsilateral ACLR (89% vs 51%). It is important for the players and for clinicians to be aware of the profound negative impact that an additional ACL injury may have on knee function and the importance of tertiary prevention.

The activity level decreased from baseline to follow-up for all groups. Between-group comparisons showed the largest decrease for players with a primary ACLR who sustained a new ACL injury. We graded activity according to the Tegner Activity Scale to compare different demanding knee activities. Previous studies comparing patients with revision^23^ or bilateral ACL injuries^8^ with patients with primary ACLR showed no difference in scores on the Tegner Activity Scale. Many players who sustain an additional ACL injury return to high knee-demanding sports after their primary ACLR and have a high Tegner Activity Score.^3^ However, their career is often short.^11^^,^^25^ Therefore, when comparing changes in activity in the same patient from primary ACLR to after sustaining an additional ACL injury the decrease in activity level could be obvious. We also included players who had additional ACL injuries but did not undergo reconstruction, which could imply decreased activity level due to persistent functional instability or other life priorities. Many female players, even players without ACLR, quit football as they get older for different reasons such as family or work commitments, lack of interest, or other reasons than the knee^11^ and therefore we also followed players with no ACL injury. At follow-up, there were significant between-group differences regarding the number who still played football. The lowest participation rate was among those who had sustained an additional ACL injury (14% still played) and players with a primary ACLR at baseline (29% still played) compared with players who were knee healthy at baseline (47% still played). These findings agree with previous studies on female football players with primary ACLR, which reported that 12%–31% were still playing at a median of seven years follow-up.^26^^,^^27^

Players with a primary ACLR who did not sustain a new ACL injury showed a slight increase in satisfaction. Between-group comparisons showed a significant decrease in satisfaction with knee function for the players with a primary ACLR who sustained a new ACL injury compared to players with a primary ACLR who did not sustain a new ACL injury and players with no ACL injury. Satisfaction with activity level decreased significantly in players with a primary ACLR who sustained a new ACL injury. In contrast, in a previous study, patients with bilateral ACL injuries did not differ compared with patients with primary ACLR regarding satisfaction with activity level.^8^ Patients who return to sport,^20^^,^^28^ have higher self-efficacy, and have greater knee-related quality of life after ACLR are more likely to be satisfied.^20^ It is important to set realistic goals after primary and especially after a second ACLR to prevent athletes’ dissatisfaction.^29^

A strength of our study is the homogeneous cohort of female football players with ACLR and without ACL injury at baseline. We had a high response rate (95%) at follow-up from players with no ACL injury, which strengthens the value of the data. The inclusion of players with no ACL injury made it possible to follow the natural course of self-reported activity level and satisfaction with knee function and activity level.^30^ When evaluating how much the players in the different groups changed from baseline to follow-up in Tegner Activity Score, IKDC-SKF, and satisfaction with knee function and activity level it is easier to get a lower follow-up score and greater changes between baseline and follow-up if you start with a higher baseline score. Therefore, we adjusted the baseline values to see if the changes depend on eventual differences in baseline values, especially between the players with primary ACLR and players with no ACL injury.

Some limitations should be acknowledged. There is a risk of recall bias regarding reports of having sustained an ACL injury during follow-up, but this risk is considered minimal because an ACL injury usually affects the player to a great extent. In addition, all new reported ACL injuries were confirmed from medical records or the SNKLR. There were few players who still played football at follow-up and therefore only descriptive football-specific data were reported and not the changes from study baseline. It is difficult to measure activity level; the Tegner Activity Scale is not a real categorical scale and includes limited types of sport even in the modified and updated score.^8^ To assign the most correct level, this was done by the first author. It could be difficult for the responders to select a level from the sparse information about sport and level of participation given in the questionnaire. Another limitation is that the two different scales used for evaluation of satisfaction are not examined for psychometric parameters. The players who sustained a new ACL injury had different follow-up times from the injury, ranging from 2 to 81 months. We performed a sensitivity analysis to account for the fact that time from injury or ACLR to follow-up can affect the results in our outcomes. Importantly, this sensitivity analysis did not change the main conclusions. Finally, we do not know if the results are valid for athletes in other sports than football and for male athletes.

## Conclusions

A primary ACL injury decreased the activity level and satisfaction with knee function. Female football players with a previous primary ACLR who had an additional ACL injury decreased their self-reported knee function, activity level, and satisfaction with knee function and activity level the most. The findings highlight the importance of primary and tertiary prevention for ACL injuries.

## Conflicts of interest

None to declare.

## References

[1] Montalvo A.M., Schneider D.K., Yut L. (2019). “What's my risk of sustaining an ACL injury while playing sports?” A systematic review with meta-analysis. Br J Sports Med.

[2] Grevnerts H.T., Kvist J., Fältström A. (2020). Patients focus on performance of physical activity, knee stability and advice from clinicians when making decisions concerning the treatment of their anterior cruciate ligament injury. Int J Sports Phys Ther.

[3] Fältström A., Kvist J., Hägglund M. (2021). High risk of new knee injuries in female soccer players after primary anterior cruciate ligament reconstruction at 5- to 10-year follow-up. Am J Sports Med.

[4] Fältström A., Hägglund M., Kvist J. (2016). Factors associated with playing football after anterior cruciate ligament reconstruction in female football players. Scand J Med Sci Sports.

[5] Cox C.L., Huston L.J., Dunn W.R. (2014). Are articular cartilage lesions and meniscus tears predictive of IKDC, KOOS, and Marx activity level outcomes after anterior cruciate ligament reconstruction? A 6-year multicenter cohort study. Am J Sports Med.

[6] Spindler K.P., Huston L.J., Wright R.W. (2011). The prognosis and predictors of sports function and activity at minimum 6 years after anterior cruciate ligament reconstruction: a population cohort study. Am J Sports Med.

[7] Chaput M., Palimenio M., Farmer B. (2021). Quadriceps strength influences patient function more than single leg forward hop during late-stage ACL rehabilitation. Int J Sports Phys Ther.

[8] Fältström A., Hägglund M., Kvist J. (2013). Patient-reported knee function, quality of life, and activity level after bilateral anterior cruciate ligament injuries. Am J Sports Med.

[9] Wright R.W., Gill C.S., Chen L. (2012). Outcome of revision anterior cruciate ligament reconstruction: a systematic review. J Bone Joint Surg Am.

[10] Goddard M., Salmon L., Waller A. (2013). Incidence of graft rupture 15 years after bilateral anterior cruciate ligament reconstructions: a case-control study. Bone Joint J.

[11] Fältström A., Kvist J., Gauffin H. (2019). Female soccer players with anterior cruciate ligament reconstruction have a higher risk of new knee injuries and quit soccer to a higher degree than knee-healthy controls. Am J Sports Med.

[12] Kvist J., Kartus J., Karlsson J. (2014). Results from the Swedish national anterior cruciate ligament register. Arthroscopy.

[13] Stein S.M., Mandelbaum B.R. (2020). Editorial commentary: anterior cruciate ligament injury and reconstruction in soccer players: the major challenge is always going for our goals!. Arthroscopy.

[14] Irrgang J.J., Anderson A.F., Boland A.L. (2001). Development and validation of the international knee documentation committee subjective knee form. Am J Sports Med.

[15] Grevnerts H.T., Terwee C.B., Kvist J. (2015). The measurement properties of the IKDC-subjective knee form. Knee Surg Sports Traumatol Arthrosc.

[16] Irrgang J.J., Anderson A.F., Boland A.L. (2006). Responsiveness of the international knee documentation committee subjective knee form. Am J Sports Med.

[17] Tegner Y., Lysholm J. (1985). Rating systems in the evaluation of knee ligament injuries. Clin Orthop Relat Res.

[18] Briggs K.K., Lysholm J., Tegner Y. (2009). The reliability, validity, and responsiveness of the Lysholm score and Tegner activity scale for anterior cruciate ligament injuries of the knee: 25 years later. Am J Sports Med.

[19] Cherkin D.C., Deyo R.A., Street J.H. (1996). Predicting poor outcomes for back pain seen in primary care using patients' own criteria. Spine (Phila Pa 1976).

[20] Ardern C.L., Österberg A., Sonesson S. (2016). Satisfaction with knee function after primary anterior cruciate ligament reconstruction is associated with self-efficacy, quality of life, and returning to the preinjury physical activity. Arthroscopy.

[21] Ardern C.L., Österberg A., Tagesson S. (2014). The impact of psychological readiness to return to sport and recreational activities after anterior cruciate ligament reconstruction. Br J Sports Med.

[22] Cristiani R., Engstrom B., Edman G. (2019). Revision anterior cruciate ligament reconstruction restores knee laxity but shows inferior functional knee outcome compared with primary reconstruction. Knee Surg Sports Traumatol Arthrosc.

[23] Grassi A., Ardern C.L., Marcheggiani Muccioli G.M. (2016). Does revision ACL reconstruction measure up to primary surgery? A meta-analysis comparing patient-reported and clinician-reported outcomes, and radiographic results. Br J Sports Med.

[24] Lefevre N., Klouche S., Mirouse G. (2017). Return to sport after primary and revision anterior cruciate ligament reconstruction. Am J Sports Med.

[25] Waldén M., Hägglund M., Magnusson H. (2016). ACL injuries in men's professional football: a 15-year prospective study on time trends and return-to-play rates reveals only 65% of players still play at the top level 3 years after ACL rupture. Br J Sports Med.

[26] Brophy R.H., Schmitz L., Wright R.W. (2012). Return to play and future ACL injury risk after ACL reconstruction in soccer athletes from the Multicenter Orthopaedic Outcomes Network (MOON) group. Am J Sports Med.

[27] Roos H., Ornell M., Gardsell P. (1995). Soccer after anterior cruciate ligament injury–an incompatible combination? A national survey of incidence and risk factors and a 7-year follow-up of 310 players. Acta Orthop Scand.

[28] Nwachukwu B.U., Voleti P.B., Berkanish P. (2017). Return to play and patient satisfaction after ACL reconstruction: study with minimum 2-year follow-up. J Bone Joint Surg Am.

[29] Feucht M.J., Cotic M., Saier T. (2016). Patient expectations of primary and revision anterior cruciate ligament reconstruction. Knee Surg Sports Traumatol Arthrosc.

[30] Ardern C.L., Taylor N.F., Feller J.A. (2012). Return-to-sport outcomes at 2 to 7 years after anterior cruciate ligament reconstruction surgery. Am J Sports Med.

